# A comparative analysis of distinct assays for the investigation of *Caenorhabditis elegans* behavior during organophosphate exposure

**DOI:** 10.17912/micropub.biology.001533

**Published:** 2025-05-16

**Authors:** Johanna Haszczyn, Vincent O'Connor, Lindy Holden-Dye, A. Christopher Green, James Kearn

**Affiliations:** 1 Biological Sciences, University of Southampton, Southampton, England, United Kingdom; 2 Defence Science and Technology Laboratory, Salisbury, England, United Kingdom

## Abstract

*C. elegans* motility is a convenient paradigm to describe the behavioral outcome of genetic- and drug-induced changes in neural circuits. Motility may be parameterized by scoring movement on solid medium or in liquid. In addition, body wall muscle contraction inhibits pharyngeal pumping, providing an indirect measure of motility. Here, the ability of these different experimental approaches to resolve organophosphate-related effects over time was investigated. In addition, two genetic mutations that alter neuromuscular function at the L-type body wall muscle were also investigated using these assays. This work highlights the benefits and limitations of distinct screening approaches for *C. elegans* behavior when analysing organophosphate mode of action on neuromuscular signalling. In particular, this work showed that pharyngeal pumping was able to resolve acute and chronic organophosphate-related effects, however liquid-based assays were best suited to resolve the phenotype of the genetic mutant L-AChR ( *ufis6).*

**Figure 1. Behavioral readouts of body wall muscle function in the face of organophosphate exposure (A-D) or a genetic gain-of-function mutation in the body wall muscle levamisole receptor (E-H):**
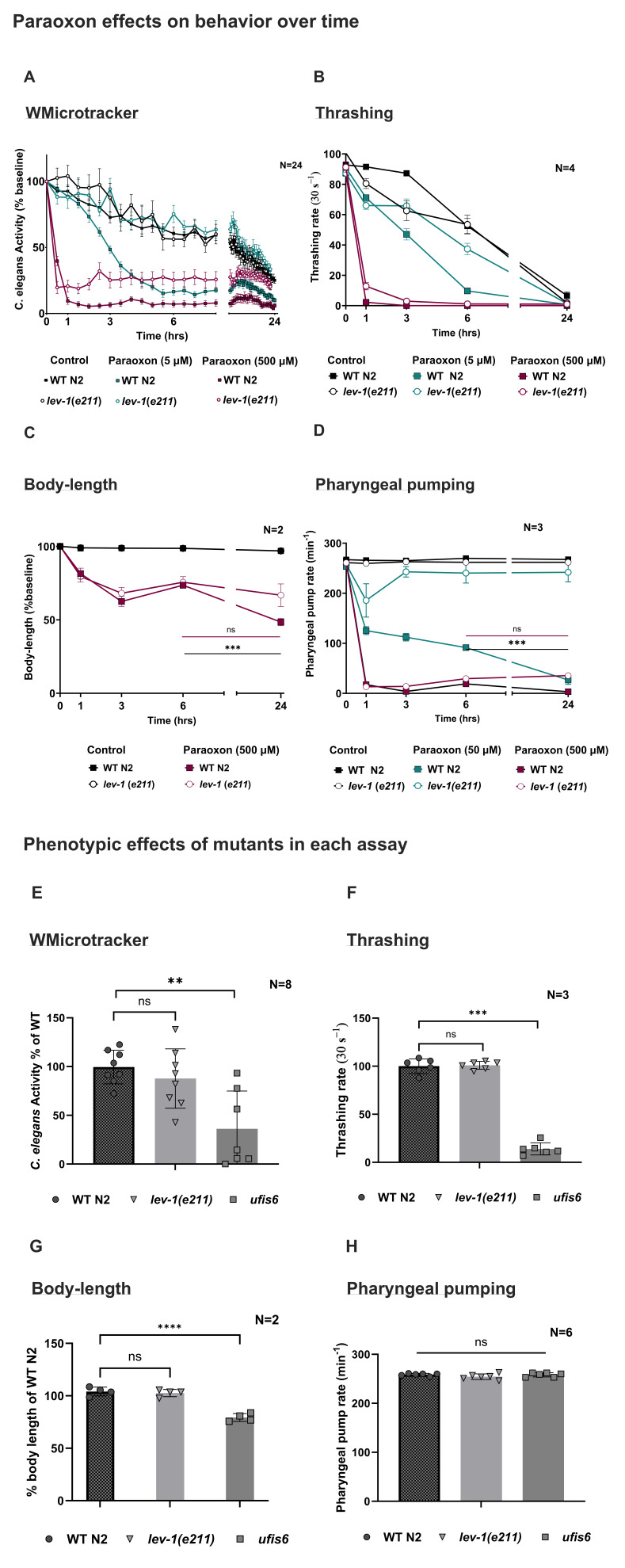
Time course effects of paraoxon on WT and mutant *lev-1* ( *e211* ) behavior in each assay (A-D) WT *C. elegans* were exposed to either control (black), an IC _50_ concentration (blue) or a supra-maximal concentration of 500 μM of paraoxon (pink) in the **A** WMicrotrackerONE motility, **B** thrashing, **C** body-length and **D** pharyngeal pumping assays. IC _50_ was calculated from a concentration-response curve of WT behavior at 3-hours in each assay to allow comparison between assays. The acute resistance of the mutant *lev-1(e211* ) was measured relative to WT at IC _50_ concentrations of paraoxon (blue) in the **A** WMicrotrackerONE motility, **B** thrashing and **D** pharyngeal pumping assays. The IC _50_ of body-length could not be measured as concentrations could not reach a 50% inhibition with acute exposure. Chronic mitigation was observed and measured at a supramaximal concentration (pink) at 6-hours in agar-based assays and the mutant effects were compared relative to WT. Phenotypic effects of genetic mutants in each assay (E-H) In the absence of drug, the mutant *lev-1* ( *e211* ) did not exhibit a background phenotype in any of the assays relative to WT behavior (E-H). The transgenic strain *L-AChR* ( *ufis6* ) exhibited a severe reduction of motility in liquid-based assays (E and F), that wasn't observed in agar-based assays (G and H). This suggests that liquid-based assays are more sensitive to mutant defects of neuromuscular function. The *lev-1(e211)* is a single amino acid change in TM4 that reduces the function of the L-type receptor. L-AChR *ufis6* is a gain of function transgenic strain expressing receptor subunits that have three amino acid changes in LEV-1, UNC-38, and UNC-63 in TM2 that cause the recombinantly expressed L-type receptor to be constitutively open. The data is presented as a mean +/- SEM. Statistical analysis was analysed with Prism 9 using two-way ANOVA with Bonferroni corrections. N is the number of assays of at least two worms. * P<0.05; **P<0.01; ***P<0.001.

## Description

Cholinergic transmission in *C. elegans* causes an excitation of muscle and controls behaviors including locomotion and feeding (Avery & You, 2012; Hughes & Vidal-Gadea, 2022; Hunt, 2017; Maglioni & Ventura, 2016; Sofela et al., 2021; Thapliyal & Babu, 2018). These behaviors can be directly measured by quantifying movement on agar or in liquid; measuring body-length and the feeding of the animal (termed pharyngeal pumping), respectively (Avery & You, 2012; Izquierdo et al., 2021). These assays have been widely deployed to parameterize drug- and mutant-related effects that alter cholinergic neurotransmission and have provided key insight into the molecular determinants underpinning neuromuscular function (Brenner, 1974; Fleming et al., 1997; Florin et al., 2023; Holden-Dye et al., 2013; Hughes & Vidal-Gadea, 2022; Hunt, 2017; Lewis, Elmer, Skimming, McLafferty, et al., 1987; Lewis, Elmer, Skimming, Mclafferty, et al., 1987; Mulcahy et al., 2013).

The aim of this study was to investigate the benefits and limitations of different assays that measure *C. elegans* motility. In recent years, higher-throughput assays have been developed, such that the movement of *C. elegans* in a liquid-based system can be used to measure both mutant- and drug-related effects on motility on a large scale with minimal labour. This high-throughput approach was used to build on our previous work that used more medium-throughput agar-based assays to assess organophosphate (OP) related effects on neuromuscular function and motility in wild-type (WT) and mutant worms (Izquierdo et al., 2021, 2022).

To assess the capabilities of *C. elegans* for OP studies, multiple assays were compared. Firstly, movement in liquid was measured using either an automated WMicrotrackerONE system or by counting the rate of thrashing through visual observation of the C-bend(s) that individual *C. elegans* perform when in liquid. This differs to the WMicrotrackerONE, which measures the frequency with which a population of *C. elegans* disrupt infrared beams that pass through the wells of a plate. Two measures were also used as a proxy for contraction of body wall muscle which impacts motility: body-length (Mulcahy et al., 2013) and the rate of pharyngeal pumping (Izquierdo et al., 2021). These distinct assays were used to resolve the effects of drugs over a time-course. During these assays, both acute and longer-term effects (described here as chronic) were observed, which relate to body wall muscle function in response to application of the OP paraoxon. In addition, the ability of the assays to measure mutant effects in the absence and presence of OP exposure was assessed. This was scored against mutants in the L-type nicotinic acetylcholine receptor (nAChR) in the body wall muscle. This receptor contributes to motility and has been thoroughly evidenced to contribute to levamisole, carbamate and OP resistance in mutants that lack or reduce receptor function (Davis et al. 2022, Chaya et al., 2021, Towers et al., 2005, Fleming et al., 1997; Florin et al., 2023; Holden-Dye et al., 2013; Hughes & Vidal-Gadea, 2022; Hunt, 2017; Lewis, Elmer, Skimming, McLafferty, et al., 1987; Lewis et al., 1987; Mulcahy et al., 2013). The use of other mutants in this receptor has found paraoxon-dependent mitigation in the presence of chronic, super-maximal concentrations (Izquierdo et al., 2023). The *lev-1 ( e211* ) and *ufis6* mutants were used as controls to measure reduced or enhanced receptor function, respectively. This comparative approach was used to investigate these different assays to assess changes in behavior during organophosphate exposure across a time course in each assay. Across the time-course, this work assessed whether the assay could measure mutant phenotypic effects, acute resistance to WT OP-inhibited behavior, but also a mitigation of behavior found in WT and mutant strains at 6-hours with longer-term exposure. This aimed to find a suitable assay to measure mutant- and OP-related effects on neuromuscular dependent behavior.

Measurement of motility in liquid either using the WMicrotrackerONE assay ( Figure 1A ) or by visual observation of thrashing ( Figure 1B ) both showed a time-dependent decrease in activity over the course of 24-hours in the unexposed WT. The decline in activity was more rapid with the WMicrotrackerONE assay compared to the thrashing assay. In the face of OP exposure both assays revealed an OP inhibition of motility. The rapid and virtually complete inhibition of activity using both assays at the high concentration of paraoxon was similar, however the inhibition of activity observed with the WMicrotrackerONE assay was not complete compared to complete inhibition of thrashing. It was suspected that the automated WMicrotrackerONE detects any movement whereas the visual observation of thrashing only scores coordinated flexion of the body by a C-bend. This discrepancy in measuring movement in liquid may suggest that motility is reduced in both assays, but the greater inhibition observed in thrashing relative to the WMicrotrackerONE suggests that the movement is uncoordinated. The gradual decline of baseline activity in both WMicrotrackerONE and thrashing assays, reported previously by other authors, suggests that movement in liquid is a measure that loses discriminatory power in our hands after 3-hours (Bianchi et al., 2015; Gunderson et al., 2020; Hahnel et al., 2021; Jones et al., 2011; Spensley et al., 2018). For this reason, the WMicrotrackerONE and thrashing assays are less useful to measure OP related effects beyond the 3-hour time point.

The decrease in motility observed in the presence of paraoxon is accompanied by a hypercontraction of body wall muscle as measured by body-length ( Figure 1C ). Body-length was used to measure hypercontraction of the worm to observe acute and chronic effects of paraoxon. An IC _50_ at 3-hours could not be calculated as a high concentration only achieved a 50% inhibition at 24-hours. Other work has used pharyngeal pumping as a tractable assay to measure OP-induced hypercontraction of body wall muscle, which we used as a read out of body-length (Izquierdo et al., 2022). In this work, pharyngeal pumping exhibited a concentration-dependent inhibition over time ( Figure 1D ). The low concentrations were measurable and achieved a 50% inhibition at 3-hours. The IC _50_ for pharyngeal pumping was greater (50 μM) than liquid-based assays (5 μM). It is suspected that this was a result of paraoxon being added to NGM for 24-hours prior to exposure in pharyngeal pumping assays, compared to paraoxon addition at the time of exposure in the liquid assay. A supra-maximal concentration was used for both body-length and pharyngeal pumping to measure high-concentration chronic effects. At the higher concentration, body-length and pharyngeal pumping exhibited a similar time-course. The body-length initially revealed an OP-induced hypercontraction in the WT evidenced by an inhibition to 60% up until 3-hours, reflected in a maximal inhibition in pharyngeal pumping. Surprisingly, at the later 6-hour time point, the body-length and pharyngeal pumping partially recovered, before a final inhibition at 24-hours. This suggests that both body-length and pharyngeal pumping are more suitable to score chronic OP effects relative to liquid-based assays. In addition, pharyngeal pumping is also able to score measurable acute effects at earlier time points relative to body-length.

Acute resistance of genetic mutants was investigated using the 0–3-hour window, where motility is retained. WT and *lev-1* ( *e211* ) worms were exposed to a WT IC _50_ concentration of paraoxon at 3-hours in all assays. The behavior of the mutant strains was compared as a percentage to the WT to measure resistance in each assay. The WMicrotrackerONE assay retained a good ability to resolve the *lev-1 ( e211* ) acute resistance, as *lev-1 ( e211* ) in the presence of an IC _50_ concentration of paraoxon did not exhibit inhibited motility relative to a 50% inhibition in the WT ( Figure 1A ). This was partially observed in thrashing however, there was less of a difference between control and OP-treated mutant worms ( Figure 1B ). This indicates that the *lev-1* ( *e211* ) mutant is still able to move in liquid, but not in a coordinated manner. This acute resistance was also replicated in the agar-based assay pharyngeal pumping ( Figure 1D ), but not in body-length ( Figure 1C ). We suspect the dynamic range of pharyngeal pumping is representative of the reduced contraction at the body wall muscle, which body-length shows a trend towards ( Figure 1C ). Altogether, our data suggests that the WMicrotrackerONE and pharyngeal pumping assays are best suited to measure acute resistance to paraoxon.

It was previously observed that chronic mitigation occurred in the presence of paraoxon in WT worms, as measured by body-length and pharyngeal pumping, at 6-hours. Importantly, the agar-based assays identified the more unexpected observation that there was an emergent mitigation of the reduced body-length and pharyngeal pumping in face of persistent and chronic exposure of drug in mutants of the *lev-1* ( *e211* ) mutant. In particular, at high concentrations this assay identified that mutants in *lev-1* can impart chronic mitigation from 6-hours up until 24-hours, which we also observed in this work ( Figure 1C and 1D). This exposure-induced mitigation of inhibition was also found to be receptor signalling organized (Izquierdo et al., 2023). The value of this is that the OP effects are revealed in face of otherwise normal WT and mutant pharyngeal pumping, and they allow difficult to resolve sub-responses in an otherwise paralysed worm where normal measures of motility provide little or no signal. The restriction of the 0–3-hour time window in the liquid-based assays limits the time in which chronic drug-dependent effects can be resolved at 6-hours in the WT background ( Figure 1A and 1B). In addition, the liquid assays failed to identify the exacerbated mitigation of drug-induced inhibition in the WT or *lev-1* mutants otherwise resolved in the pumping and body-length assays on agar ( Figure 1A and 1B). This resolution is confounded by either i. the assays in solution failing to express the phenotype or ii. the loss of dynamic range and the fall in control motility after 3-hours. Altogether, this suggests that liquid-based assays are unsuitable for investigating chronic OP-related effects on both WT and mutant backgrounds, which can be resolved using pharyngeal pumping. This suggests that pharyngeal pumping may be a useful addition to OP/genetic investigations that shape acute and chronic drug effects to inform mode of action, neural plasticity and underpinning of toxicology and resistance (Izquierdo et al., 2021, 2022, 2023).

Although the liquid motility failed to resolve important OP-induced effects, it did reveal phenotypes associated with genetic mutations not identified in the pharyngeal pump assay ( Figure 1E- H). The ability to resolve otherwise stealth phenotypes is highlighted by analysis of gain–of-function lines expressing hyper-functional body wall muscle receptors (L-AChR *(ufis6* )). This strain appears normal in their pumping and has a subtle change in on food motility (Bhattacharya et al., 2014). This highlights that the ability to resolve genetic- and OP-dependent changes in motility is dependent on the assay used. To highlight this effect, we have indicated if the selected assay can resolve genetic ( Figure 1E- H), acute and chronic OP effects ( Figure 1A- D) in the figure above to direct where the pros and cons of each assay lies.

**Conclusion**

In this work, we have compared agar-based and liquid-based assays to assess OP and mutant effects on the body wall neuromuscular function. The different methods assessed OP-dependent acute and chronic effects on behavior that may be pertinent in other analysis of acute and chronic effects of OPs read out through neuromuscular junction. This suggests that the selection of bioassay has important consequences for biological read out. This echoes earlier studies that resolved how chronic exposure of distinct classes of cholinergic drugs resolve differing concentration- and time-dependent effects that resolve both drug mediation and modulation. As highlighted here the more modulatory effects that follow the initial acute engagement or drug receptors require assay formats that sustain the organism and the control readouts against which supporting modulation can be scored. This highlights that pharyngeal pumping is a useful bioassay as a readout of OP induced body wall muscle hypercontraction and recovery, but also the value of liquid-based assays to measure phenotypic measurements.

## Methods

***C. elegans* maintenance**

N2 Wild-type *C. elegans* strain obtained from Caenorhabditis Genetics Center (CGC) were maintained under standard conditions (Brenner, 1974). *C. elegans* were grown at 20°C on Nematode Growth Medium (NGM) agar plates seeded with *E. coli* OP50 as a source of food. The strains *lev-1 ( e211* ) was acquired from the CGC. The transgenic line IZ236 ufIs6 [ *Pmyo-3:: unc-38 (V/S* ), *Pmyo-3:: unc-29 (L/S)* , *Pmyo-3:: lev-1 (L/S)* ] was kindly provided by Francis Lab (UMass Chan Medical School).

**Drug stocks**

1M Paraoxon-ethyl (Merck) dissolved in DMSO. A 100% DMSO stock was diluted into assay agar or liquid such that vehicle concentration was no higher than 0.1% (Calahorro et al., 2021). The drug stocks were kept at 4 ᵒC.

**Behavioral assays for motility and/or body wall muscle function**

All behavioral experiments described here were performed on a standard developmental stage L4 + 1 day young adults at room temperature (20ᵒC). *C. elegans* were viewed under a Nikon SMZ800 binocular zoom microscope. L4 worms were recognised based on the vulva saddle, which were selected 16−24 hours before the experiment and placed on fresh *E. coli* OP50 seeded plates.

Thrashing

Thrashing was counted as previously described. For control and OP exposed groups, thrashes were counted at indicated times for 30 seconds. Any change in the midbody bending direction was referred to as a thrash (Miller et al., 1996). L4+ 1 adults were removed and placed in 100 ml M9 of a 96-well plate, left for 30 seconds to adapt and then thrashing was measured for 30 seconds. L4+1 worms were prepared either by picking or through bleaching protocol used in the WMicrotrackerONE assay.

WMicrotrackerONE

The WMicrotrackerONE measures worm movement through infrared-based tracking.

A bleaching protocol was used to make a synchronised population of synchronised L4 + 1/adult *C. elegans* (Hibshman et al., 2021). Each plate contained five gravid adults that were left for three days before bleaching. This generated around 10,000 worms from fifteen 9 cm well plates. These were washed off into a falcon tube and washed three times with M9 in a total volume of 15 ml and on the final wash the supernatant was removed until 3 ml. Then, 10 μl of this solution was pipetted into each well of a 96 well plate. Number of worms per well (typically 10-15 worms per well) were counted by visual inspection and recorded for normalisation. Total volume in each well was 100 μl (80 μl M9, 10 μl worms and 10μl drug/vehicle). The plate was added into the WMicrotrackerONE to record the baseline level of *C. elegans* activity (AU) for 30 minutes before 1000-fold drug stocks were added into each well. The assay was left to run for 1400 minutes. Data was normalised to the number of worms in each well. The *C. elegans* activity was then plotted as a percentage of the baseline activity taken from 0-30 minutes.

Estimates of IC _50_ were made by measuring the *C. elegans* activity at indicated drug concentrations after a 3-hour incubation relative to worms placed on drug free vehicle control plates. The percentages of inhibition relative to these controls were used to measure the IC _50_ .

Pharyngeal pumping

Pharyngeal pumping on food was scored at indicated times after transferring worms to drug or vehicle control plates. *C. elegans* that left the patch of food during the experiment were picked back to the bacterial lawn and their pump rate recorded 10 minutes after return to food. Pumping was quantified by counting the number of grinder movements observed under binocular microscope using a counter. In our experiments we note that in reduced drug inhibited pumping is more varied therefore the pump rate was quantified for a minimum of 2 minutes per worm at each time point and plotted as pumps per minute.

The estimates of IC _50_ were made by measuring the pharyngeal function at indicated drug concentrations after a 180-minute incubation relative to worms placed on drug free vehicle control plates. The percentages of inhibition relative to these controls were used to measure the IC _50_ .

Body-Length

The body length was measured as previously described (Izquierdo et al., 2021; Mulcahy et al., 2013). Briefly, images of the worms were acquired through a Nikon SMZ800 binocular zoom microscope. These images were binarized and skeletonized using ImageJ software. The length of the skeleton was used to determine the body length of the nematodes. % inhibition was calculated relative to WT control at time 0.

**Plate Husbandry**

Agar plates

Experiments were performed in 6-well plates containing a final NGM volume of 3 ml as previously reported (Izquierdo et al., 2023). Organophosphate-containing plates were prepared the day before by adding an aliquot of a concentrated drug stock into the melted NGM. 50 μl of *E. coli* OP50 bacteria was added on the plate when the media was solidified. After 1 hour in a fume cupboard, the dried bacterial plates were sealed and kept in dark at 4 ᵒC until next day.

Plates were left at room temperature for at least 30 minutes before starting the experiment. Neither vehicle concentration alone had any effect in the phenotypes tested.

Liquid plates

Experiments were performed in 96-well plates containing a final M9 volume of 100 μl. Worms were picked directly from an unseeded plate into each well. A 10 μl aliquot of a concentrated stock of vehicle or organophosphate was added to plates already containing 90 μl M9 after the baseline recording in the WMicrotrackerONE.

**Statistical analysis**

All statistical analyses were performed using GraphPad Prism 9 and are displayed as the mean ± SD. Statistical significance was measured using either a one-way or two-way ANOVA test followed by Bonferroni post hoc analysis. Bonferroni corrections were selected to avoid false positives. The sample size N of each experiment is specified in the result figures.

## Reagents

**Table:** 

Strain	Genotype	Source
CB211	*lev-1* ( *e211* )	CGC
IZ236	ufIs6 [ *Pmyo-3:: unc-38 (V/S* ), *Pmyo-3:: unc-29 (L/S)* , *Pmyo-3:: lev-1 (L/S)* ]	Francis Lab, UMass Chan Medical School
